# The study on the risk of other endocrine glands autoimmune diseases in patients with type 1 diabetes mellitus

**DOI:** 10.1097/MD.0000000000020437

**Published:** 2020-05-29

**Authors:** Yang Liu, Shuchun Chen, Dongmei Zhang, Zelin Li, Xing Wang, Xing Xie, Haijiao Zhu, Luping Ren, Liqin Wang

**Affiliations:** a Department of Endocrinology, Hebei General Hospital; b Department of Endocrinology; c Key Laboratory of Environment and Population Health of Hebei Province, Department of Epidemiology and Statistics, Hebei Medical University, Shijiazhuang, Hebei, PR China.

**Keywords:** Addison disease, antibody, autoimmune polyendocrine syndromes, autoimmune thyroid disease, hypogonadism, hypoparathyroidism, type 1 diabetes mellitus

## Abstract

To study the changes of pancreas, thyroid, adrenal, parathyroid and gonadal organ-specific antibodies in patients with type 1 diabetic patients and to explore the risk of development to other endocrine gland autoimmune diseases.

Fifty one patients with type 1 diabetes mellitus were selected. ELISA was used to detect islet, adrenal gland, Parathyroid, gonadal organ-specific antibody levels, the level of thyroid-related antibodies by lectrochemiluminescence.

Compared with the healthy control group, the levels of the 17-α-OHAb, 21-OHAb, NALP5Ab, P450sccAb, and CaSRAb in the T1DM group were significantly higher. GADAb-positive patients were more likely to have TPOAb-positive patients than GADAb-negative patients, and the positive rate of 2 thyroid antibodies in GADAb-positive patients was significantly higher than that in GADAb-negative patients. The presence of these antibodies is related to the age of onset of type 1 diabetes or Patient age. In combination with 1 or 2 islet antibody-positive patients, the combined non-islet antibody positive rate was higher than that of islet antibody-negative patients.

Patients with type 1 diabetes with other autoimmune diseases at risk significantly increased compared with normal, of which the most common thyroid autoimmune disease, thyroid antibodies and hormone levels should be routinely detected at the first visit and long-term follow-up.

## 1 Introduction

Type 1 diabetes mellitus (T1DM) is the most common chronic disease in the minors. There is a significant difference in the incidence rate in different countries. The statistical results in China show that the incidence of this disease is about 0.1/100,000 per year.^[1]^ Despite the low incidence, the occur of T1DM in children worldwide is increasing at a rate of 3% to 5% per year, which has grown up to be an important risk factor affecting the physical and mental health of minors in China.^[2]^ The pathogenesis of T1DM is complicated and diverse. At present, considering the combination of environmental factors, immune factors, and genetic factors, the bodys own immune response destroys islet cells, leading to insulin deficiency. Studies have shown that about one-third of patients with T1DM develop autoimmune responses in islets, while other organs also produce immune damage. When more than 2 endocrine organs are damaged, they develop autoimmune multiple Autoimmune polyendocrine syndromes (APS). In 1980, Neufeld and Blizzard divided them into 4 types according to the pathogenesis and clinical manifestations of APS: APSI: adrenal insufficiency, hypoparathyroidism, and chronic mucosal cutaneous candidiasis at least 2 diagnosed this disease. APSII also known as Schmidt syndrome. Adrenal insufficiency combined with T1DM and autoimmune thyroid diseases (AITD) can be diagnosed. APSIII, except for adrenal insufficiency, hypoparathyroidism and candidiasis, AITD combined with at least one other autoimmune disease, further divided into 4 categories according to its phenotype: APSIIIA is AITD combined with other Immune endocrine gland disease, type IIIB is AITD combined with autoimmune gastrointestinal disease, type IIIC is AITD with autoimmune nervous system, skin disease, and type IIID is AITD combined with rheumatic immune disease. APSIV: when APS patients can not meet the above 3 types of classification can be classified as this type.^[3]^ At present, the pathogenesis of APS is still unclear. Only the APSI has a clear autoimmune regulator gene (AIRE) pathogenic gene, and the rest of the classification is mainly based on the clinical manifestations of the disease.

As a product of autoimmune response, autoantibodies tend to rise before clinical manifestations, so the detection of related autoantibodies has a very high predictive and diagnostic value for the occurrence of autoimmune diseases. In recent decades, significant progress has been made in the study of autoantibodies involved in autoimmune processes in autoimmune patients. Many receptors, enzymes and hormones have been identified as target antigens for organ-specific autoimmune diseases, providing a large basis for the diagnosis of APS.^[4]^ Currently, islet-specific autoantibodies include glutamic acid decarboxylase antibody (GADAb), protein tyrosine phosphatase antibody (IAA-2), anti-islet cell antibody (ICA), anti-insulin antibody and anti-zinc transporter 8 antibody. Thyroid-related antibodies include Graves disease-specific antibody: thyrotrophin receptor antibody (TRAb) and autoimmune thyroiditis-specific antibodies: thyroid peroxidase antibody (TPOAb), thyroglobulin antibody (TGAb). At the same time, including adrenal specific antibody: 21-hydroxylase antibody (21-OHAb), gonadal specific antibody: 17-α-hydroxylase antibody (17-α-OHAb), P450 side chain cleavage anzyme antibody (P450sccAb), and parathyroid specific antibodies: calcium-sensing receptor antibody (CaSRAb) and NACHT leucine-rich-repeat protein 5 antibody (NALP5Ab) have also been gradually discovered by the majority of scholars. Although the above indicators are not currently used in clinical practice, the detection of serum levels provides strong evidence for the risk study of T1DM patients with other autoimmune diseases.

The aim of this study was to evaluate the risk of autoimmune damage in T1DM patients with other organs by detecting autoimmune-related antibodies in other endocrine glands of patients with T1DM, and to provide evidence for early detection and prevention of APS.

## 2 Research object and method

### 2.1 Research object

T1DM group: The study subjects were patients with T1DM who were treated in the outpatient department and ward of the Hebei General Hospital from June 2017 to January 2018. All of them met the 1999 WHO diagnostic criteria for diabetes, a total of 51 cases, 19 males and 32 females, aged between 3 and 63 years old. Healthy group: the study subjects were from June 2017 to January 2018 in the physical examination center of Hebei General Hospital for physical examination, a total of 100 cases, 34 males, 66 females, aged 7 to 62 years, had no endocrine and autoimmune diseases and family history, and excluded those with severe liver and kidney disease, recent serious infections, trauma, or other stressful conditions. The subjects and their families were informed of the study and signed informed consent, and were discussed and approved by the hospital's academic ethical committee.

### 2.2 Research methods

All the subjects completed a questionnaire designed by professionals to record name, gender, age, waist circumference, height, weight, T1DM diagnosis time, whether or not combined with other autoimmune diseases and confirmed time, family history, past history, and so on; After 8 to 10 hours of fasting, the 2 groups of subjects took 5 ml of venous blood in the early morning of the next day, collected in a coagulation tube, allowed to stand at room temperature for 30 minutes, centrifuged at 3000 rpm for 20 minutes, and took the upper serum to 1.5 ml. The serum were stored in an Eppendorf tube at −80° C in a freezer. GADAb, IAA-2, 17-α-OHAb, 21-OHAb, NALP5Ab, P450sccAb and CaSRAb were detected by ELISA, and TRAb, TPOAb and TGAb were detected by electrochemiluminescence method.

### 2.3 Antibody positive determination method

According to the instruction manual of the kit, GADAb and IAA-2 ≥ 10 IU•ml^−1^ are defined as positive, TRAb ≥ 1.75 IU•L^−1^, TPOAb ≥ 34 IU•ml^−1^, and TGAb ≥ 115 IU•ml^−1^ is positive. The normal upper limit of 17-α-OHAb, 21-OHAb, NALP5Ab, P450sccAb, and CaSRAb was calculated using the mean antibody index + 3SD of 100 healthy individuals.^[5]^ Any serum with an antibody index above the upper limit of normal was designated as autoantibody-positive.

### 2.4 Statistical processing

Data were analyzed using SPSS version 21.0. The normal distribution measurement data is expressed by the mean ± standard deviation, and the non-normal distribution measurement data is expressed by the median (IQR). *T* test was used when the mean of 2 samples was in normal distribution with homogeneous variance, and rank sum test was used for non-normal distribution or unequal variance. Whether there is a statistical difference between 2 or more population rates or whether there is a difference between the categorical variables by the following methods: If n ≥ 40 and T ≥ 5, choose the Pearson Chi-Squared, if n ≥ 40 and 5 >T ≥ 1, choose continuous correction, if n < 40 or T < 1, choose Fisher exact test. Statistical significance was *P* ≤ .05.

## 3 Result

### 3.1 Comparison of general data between T1DM group and healthy group

Differences in sex, age, abdominal circumference, and body mass index between the 2 groups were compared by *t* test or rank-sum test. The results are shown in Table 1. There was no significant difference in sex and age. Compared with the healthy control group, the waist circumference and body mass index of the T1DM group were significantly lower than those of the healthy control group.

**Table 1:**
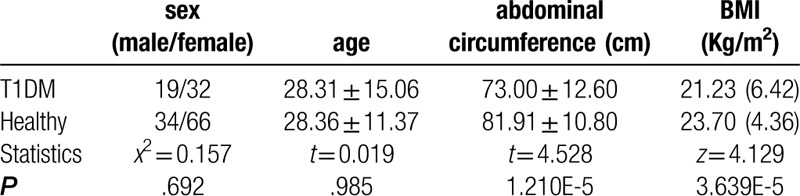
Comparison of general data between T1DM group and healthy group.

### 3.2 Clinical manifestations and combined APS status in patients with T1DM

#### 3.2.1 Clinical manifestation

Counting the past medical history of 51 patients, a total of 19 patients (37.25%) had clinical disease, and 16 patients (31.37%) with thyroid autoimmune disease. Among them, 9 patients (17.65%) were Graves disease, and the patients had different degrees of palpitations, hand tremors, sweating, and other symptoms. 7 cases (13.73%) with Hashimotos thyroiditis, patients with varying degrees of fatigue, chills, and other symptoms in the later stages of the disease. The remaining 3 patients (5.88%) with subclinical hypothyroidism. There was 1 patient with hypoparathyroidism (1.96%), 1 patient (1.96%) with Addison disease, 3 patients (5.88%) with vitiligo, and 1 patient (1.96%) with alopecia.

#### 3.2.2 Patients combine with APS

According to the clinical manifestations, 51 patients were further identified by APS classification. One case (1.96%) of T1DM patients with Graves disease, Addison disease, and hypoparathyroidism were classified as APSI. Twelve T1DM patients (23.53%) with thyroid autoimmune disease and were classified as APSIIIA. Three T1DM patients (5.88%) combined with Graves disease and vitiligo were classified as APSIIIC. No APSII patients were found in this study. There was no significant correlation between the incidence of APS and the sex, age, duration, age at diagnosis.

### 3.3 Comparison of 17-α-OHAb, 21-OHAb, NALP5Ab, P450sccAb, and CaSRAb levels in T1DM group and healthy group

The *t* test and rank-sum test were used to compare the antibody levels of the 2 groups. The results are shown in Table 2. The levels of 17-α-OHAb, 21-OHAb, NALP5Ab, P450sccAb, and CaSRAb were significantly higher in the T1DM group than in the healthy group.

**Table 2:**
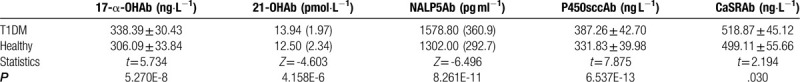
Comparison of other antibodies levels in T1DM group and healthy group.

### 3.4 Organ specific antibody positive in T1DM group

There were 34 patients (66.67%) with positive insulin antibody, 31 patients (60.78%) with GADAb positive, and 16 patients (31.37%) with IAA-2 positive. Twenty seven T1DM patients (52.94%) were positive with other endocrine gland antibodies, including 24 case of TPOAb-positive patients (47.06%), 10 cases of TGAb-positive patients (19.61%), 9 cases of TRAb-positive patients (17.65%), and 2 cases of NALP5Ab-positive patients (3.92%), 3 cases of P450sccAb positive patients (5.88%), 21-OHAb, 17-α-OHAb and CaSRAb positive patients were not found.

### 3.5 Correlation analysis of sex, age, duration, age of diagnosis, and antibody positive in T1DM group

Patients in the T1DM group were divided into 2 groups according to median in general. The relationship between general conditions and islet, thyroid, parathyroid, gonadal antibodies was analyzed by Pearson Chi-Squared, continuity correction and Fisher exact test. According to the median age of diagnosis, the patients in the T1DM group were divided into 2 groups. The positive rate of TPOAb was significantly higher in patients with confirmed age >18 years than those with confirmed age ≤18 years old. It was found that patients with age ≤28 years had an increased incidence of islet antibody compared with older patients, but patients with age >28 years were more likely to have TGAb. According to the median duration of diabetes, patients in the T1DM group were divided into 2 groups. GADAb was more positive in patients with duration ≤5 years than in patients with duration >5 years. There was no significant correlation between the duration of diabetes in the T1DM group and the islet, thyroid. TGAb and TRAb positive have nothing to do with the general conditions of patients with diabetes. Patients were grouped according to the number of insulin and thyroid antibody positives, and their relationship with the general conditions was analyzed using Pearson Chi-Squared, continuity correction and Fisher exact test. The female patients with T1DM group had a higher positive rate of 2 islet-antibody than male. The probability of 2 thyroid antibody positive cases was also significantly increased in patients diagnosed with age> 18 years. It was found that patients with age ≤28 years had an increased incidence of islet antibody compared with older patients, but patients with age >28 years were more likely to have 2 thyroid antibodies. According to the median duration of diabetes, patients in the T1DM group were divided into 2 groups. There was no significant correlation between the duration of diabetes in the T1DM group and the number of islet and thyroid antibodies. This study also found no significant association between general conditions and parathyroid, gonadal-related antibodies. One and 3 thyroid-antibodies positive have nothing to do with the general conditions of patients with diabetes.

### 3.6 Correlation between islet-associated antibodies and non-islet antibody positive in T1DM group

The Pearson Chi-Squared test and continuity correction was used to analyze the correlation between GADAb, IAA-2 antibody levels and thyroid, parathyroid, gonadal antibody positive results. As shown in Table 3, the incidence of TPOAb in GADAb-positive patients was higher than that in GADAb-negative patients, GADAb was not found to be significantly associated with TGAb, TRAb, parathyroid and gonad antibody positive. As shown in Table 4, the positive rate of 2 thyroid antibodies in GADAb-positive patients was significantly higher than that in GADAb-negative patients. GADAb was not found to be significantly associated with 1 thyroid antibodies, 3 thyroid antibodies, parathyroid and gonad antibody positive. This study also found no significant association between IAA-2 and thyroid, parathyroid, gonadal antibody positivity.

**Table 3:**
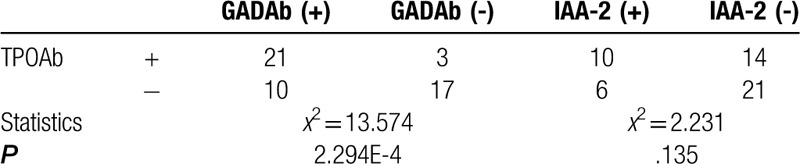
Correlation between islet-associated antibodies and TPOAb in T1DM group.

**Table 4:**
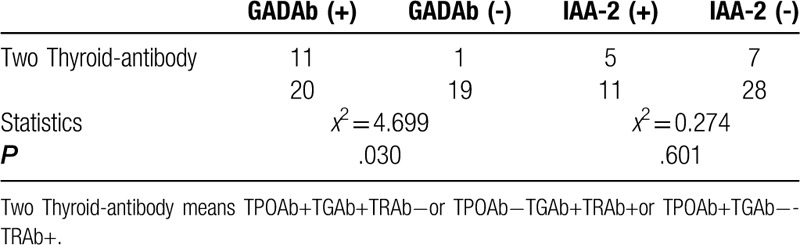
Correlation between islet-associated antibodies and 1 Thyroid-antibody positive in T1DM group.

### 3.7 Correlation between non-islet antibody positive rate, and positive number of islet antibody

**Figure 1:**
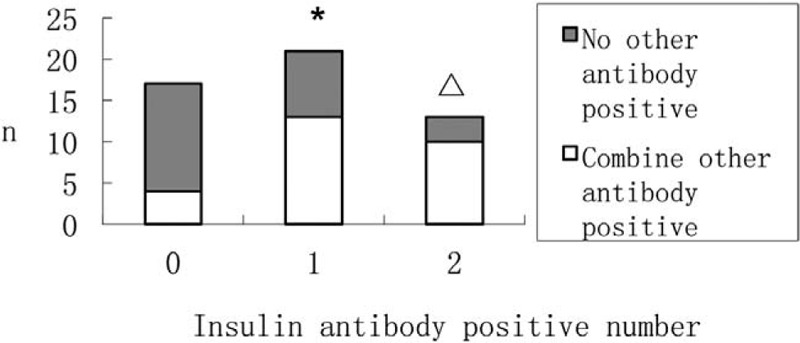
Correlation between non-islet antibody positive rate and positive number of islet antibody. ^∗^*P* < .05 compared with group 0, △*P* < .05 compared with group 0.

According to the positive number of islet antibody, the subjects in the T1DM group were divided into 3 groups: 0, 1, and 2. The Person Chi-Squared test was used to analyze the correlation between the number of islet antibody positive and islet antibody-positive patients with non-islet antibody positive rate. The results are shown in Table 5 and Figure 1. In combination with 1 or 2 islet autoantibody-positive patients, the combined non-islet antibody positive rate was higher than that of islet antibody-negative patients.

**Table 5:**
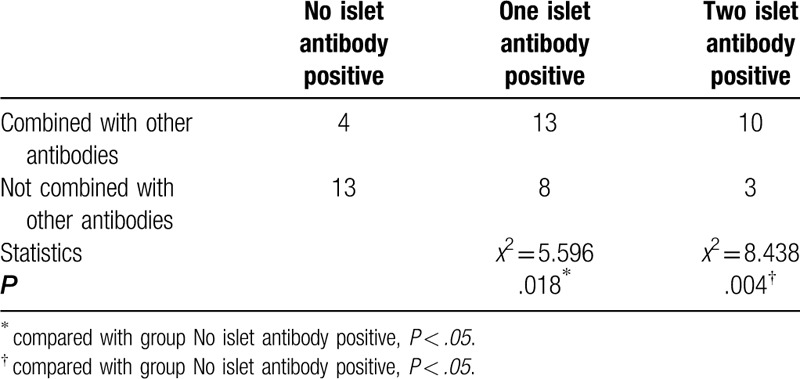
Correlation between non-islet antibody positive rate and positive number of islet antibody.

## 4 Discussion

GADAb is an antibody produced by cross-immunization of the body. It has high sensitivity, specificity and long duration. The positive rate in T1DM patients is about 70% to 80%. It is a primary autoimmune indicator commonly used in clinical evaluation to determine whether or not to combine T1DM. IAA-2 is a type I transmembrane mucin, an important component of islet cell antibodies, with high sensitivity and specificity, and a positive rate of 72% in newly diagnosed T1DM patients. Studies have shown that subjects with multiple insulin autoantibodies are more likely to have a higher risk of future T1DM than those with only 1 autoantibody. Therefore, multiple islet autoantibodies combined detection can improve the diagnostic rate of T1DM and the predictive value of T1DM.^[6]^ The study aimed to find a link between T1DM and the risk of autoimmune diseases in other endocrine glands, so the combination of serum concentration levels of GADAb and IAA-2 with high sensitivity and specificity was selected. In this study, 34 patients with positive insulin antibody accounted for 66.67%, 31 patients with GADAb positive, accounting for 60.78%, and 16 patients with IAA-2 positive, accounting for 31.37%, indicating that the positive rate of insulin antibody combined detection was significantly higher than that of single insulin antibody, which is consistent with the above conclusions. Female patients with a positive rate of GADAb and IAA-2 antibodies were significantly higher than men, which was consistent with previous studies.^[7]^ In this study, patients with age ≤28 years had an increased incidence of islet antibodies compared with older patients, which may be related to insulin antibody partial necrosis in patients with longer duration of diabetes. The study also found that GADAb-positive patients with a TPOAb positive rate and a positive rate of both thyroid antibodies were significantly increased. In combination with 1 or 2 islet antibodies, the positive rate of autoantibodies in other glands was significantly higher than that in pancreatic autoantibodies. Therefore, it is recommended that clinicians should be alert to the positive insulin antibody, especially the risk of other autoimmune diseases in patients with positive multi-insulin antibodies.

Autoimmune thyroid disease is the most common autoimmune disease in T1DM patients. About 15% to 30% of patients with T1DM can be combined with AITD. The incidence rate is 2 to 4 times that of normal people, and most of them occur after T1DM.^[8]^ According to Kordonouri et al, the most common case is Hashimotos thyroiditis, the incidence rate can reach 14% to 28%, and the incidence of Graves disease is less, ranging from 0.5% to 7%.^[9]^ The incidence of Hashimotos thyroiditis in this study was 13.73%, which was consistent with the above studies, but the incidence of Graves disease was 17.65%, which was significantly higher than the above study. Consideration was related to Chinese peoples high iodine diet and the small sample size of this study. It has been pointed out that the incidence of anti-thyroid antibodies in patients with T1DM increases with age, duration of disease, and the level and duration of GADAb.^[10]^ This study examined TPOAb, TGAb, and TRAb in patients with type 1 diabetes. The positive rate of TPOAb and the positive rate of 2 thyroid antibodies were significantly higher in patients with diagnosed age >18 years. Patients with age >28 years were more likely to have TGAb and 2 thyroid antibody positives, which is consistent with the above studies. The results of this study showed that GADAb-positive patients with TPOAb-positive or combined 2 thyroid antibody-positive chances were significantly higher than GADAb-negative, which were also consistent with the above studies. The mechanism of thyroid disease associated with GADAb may be due to the expression of glutamate dehydrogenase in thyroid follicular cells, which induces the conversion of glutamate to γ-aminobutyric acid, thereby participating in the regulation of thyroid hormone secretion. Although AITD is a common disease in China, the incidence of hypoglycemia, acute diabetic complications, and cardiovascular risk in patients with T1DM and AITD is significantly higher than that of normal people. Thyroid hormone enhances intestinal absorption of glucose, glycogenolysis, and catabolism of insulin in the liver, which can lead to hypoglycemia in patients with hypothyroidism and ketoacidosis in patients with hyperthyroidism.^[11,12]^ Subclinical hypothyroidism in patients with T1DM can lead to increased blood lipids. Therefore, early detection and treatment can reduce the risk of acute diabetic complications, hyperlipidemia, and atherosclerotic cardiovascular disease.^[13]^ The International Association for Child and Adolescent Diabetes has recommended in early 2009 that thyroid antibodies (TGAb, TPOAb) and hormone levels should be routinely screened at the time of T1DM initial diagnosis and reviewed every 2 years.^[14]^

The incidence of Addison disease (AD) in patients with T1DM is 1% to 2%, and the incidence of adrenal specific antibodies is 0% to 4%.^[2]^ There are statistics showing that 84% of patients with at least 1 adrenal specific antigen positive in AD patients, and 21-OHAb is the main marker antibody of AD.^[15]^ 21-OHAb-positive patients were not included in the study, but 1 patient enrolled in the group had a history of AD for 6 years, which may be associated with longer duration of AD in this patient, 21-OHAb turned negative and related to antibody sensitivity to the diagnosis of disease. Related studies have also proposed that the positive rate of adrenal antibody detection by immunofluorescence is higher, our study using Elisa to detect adrenal antibodies may also be one of the 21-OHAb negative causes.^[16]^ 17-α-OHAb, P450sccAb can also occur in patients with hypogonadism, positive results of these 2 indicators alone cannot play a role in the diagnosis of AD, but positive results of 21-OHAb combined with these 2 indicators are a sign of Addisons combination with other multi-glandular autoimmune diseases. Although the 21-OHAb was not found in the patients enrolled in this study, the concentration of 21-OHAb in the experimental group was significantly higher than that in the healthy control group, indicating that patients with T1DM still have a higher risk of AD, which requires the vigilance of the clinician. At present, there is no guideline for the diagnosis and treatment of T1DM combined with AD. It is not recommended to routinely detect adrenal-related antibodies in clinical practice. Therefore, it is not possible to predict the occurrence and risk of adrenal autoimmune disease in time. In China, the diagnosis of AD is mainly based on the determination of cortisol levels and the clinical manifestations of cortisol deficiency, while glucocorticoid deficiency leads to increased insulin sensitivity, increased glycolysis, glycogen production, and gluconeogenesis, resulting in recurrent hypoglycemia in AD patients, so repeated unexplained hypoglycemia symptoms can promote the diagnosis of AD in patients with type 1 diabetes.^[17]^ Studies have suggested that patients with T1DM over 18 years of age should have timely detection of cortisol hormone levels and rhythms if clinical adrenal immune disease symptoms (such as hypotension, hypoglycemia, weight loss, nausea, whitening of the skin mucosa, etc.), if the first-degree relative of a T1DM patient develops the disease, the patient should be screened every 5 years.^[16,18]^

The study of T1DM with hypogonadism is not common. Kota et al performed a follow-up of 260 patients with T1DM for 14 years and found that 5.4% of patients with T1DM developed hypogonadal disease.^[19]^ Autoimmune hypogonadism is common in women, with a male to female ratio of 1:3, which may be related to the male-specific blood testis barrier that protects the testes from autoantibody damage, there are also studies suggesting that this may be related to female pregnancy, ovarian secretion of hormones, and (or) the genetic effect of the second X chromosome.^[20]^ Gonadal-specific antigens include 2 steroidogenic enzymes: 17-α-OH and P450scc. The levels of 17-α-OHAb and P450sccAb in the T1DM group were significantly higher than those in the healthy group, but no 17-α-OHAb-positive patients were found. Two female patients and 1 male patient were positive for P450sccAb, but these 3 patients did not show a corresponding change in clinical manifestations and hormone levels of hypogonadism. Some studies have found that the positive rate of thyroid and pancreas-specific antibodies in patients with positive goiter-specific antibodies is significantly higher.^[21,22]^ Our study did not find above correlation, but 2 female patients with positive P450sccAb were positive for thyroid antibody, 1 of them combined with Hashimotos thyroiditis. Patients with hypogonadism have similar autoantibodies to adrenal antibodies, so hypogonadism often occurs with AD. Studies have shown that about 20.2% of patients with AD can be associated with hypogonadism.^[23]^ Despite the low incidence of the disease, it reduces the reproductive function of patients and seriously affects the quality of life of patients. Therefore, when T1DM patients have symptoms such as slow growth, sparse hair, genital dysplasia, amenorrhea, etc., detect sex hormone levels early, and then early detection and intervention to improve patient outcomes.

Hypoparathyroidism (HP) is one of the 3 major diseases that make up APSI. Although HP can occur in APSII-IV in some case reports, the current literature confirms that this disease only occurs in the APSI,^[24,25]^ and the incidence of T1DM in APSI patients is only 4% to 18%,^[2]^ so the incidence of this disease in T1DM is extremely low, there is no relevant literature to calculate its specific incidence. Two parathyroid specific autoantibodies, CaSRAb and NALP5Ab, have been discovered in the current study. This study examined the above 2 indicators in the enrolled patients. The levels of CaSRAb and NALP5Ab in the T1DM group were significantly higher than those in the healthy group, indicating that the risk of HP in T1DM patients was higher than that in normal subjects, and 2 of those patients were positive for NALP5Ab. Soderbergh et al did not observe CaSRAb in 73 patients with APSI with hypoparathyroidism.^[15]^ Kemp et al did not find CaSRAb expression in 178 APS patients with autoantibody screening.^[5]^ In this study, no CaSRAb positive patients were found in the T1DM group. This may be due to CaSRAb is mainly expressed in the APSI patients, the positive rate in other types of APS is only 2.2%, and the antibody specificity of diagnosis of HP and sensitivity is low, at 83% and 39% respectively, these reasons could cause CaSRAb is not easy to express in patients with T1DM.^[26–28]^ NALP5Ab only occurred in the APSI patients and has 100% and 49% specificity and sensitivity for HP diagnosis in APSI patients. This study has a NALP5Ab positive patients with AD and HP, consider for the APSI, via detecting the patients NALP5Ab positive, further proves the NALP5Ab sensitivity is higher, is the first choice for screening for HP.^[29,30]^

In addition to the above diseases, T1DM can also be combined with non-secretory gland autoimmune diseases, such as 11% of patients with celiac disease, 6.5% of patients with autoimmune gastritis, 3% to 30% of patients with autoimmune hepatitis, 0.5% to 1% of patients with vitiligo, etc., 0.19% of patients can be complicated by adolescent idiopathic arthritis.^[2,31]^ In this study, 3 patients with vitiligo, including 1 patient with alopecia, these autoimmune diseases have a common susceptibility gene associated with T1DM patients, so T1DM patients should be alert to other non-endocrine gland lesions.

The APSI type is a rare autosomal recessive disorder caused by a mutation in the AIRE gene located on chromosome 21q22.3, first reported by Leonard in 1929.^[3]^ The incidence of APSI is rare, about 1:100 000.^[32–34]^ This disease is often caused by babies, more common in women, usually with Candida albicans infection as the primary symptom, and HP is the most common, the incidence rate is 80% to 85%, followed by AD, the incidence rate is 60% to 70%, other Endocrine gland diseases include: T1DM incidence is 4% to 18%, 12% of patients with hypogonadism, 10% of patients with AITD.^[35]^ Currently screening specific antibodies and AIRE genes are the diagnostic methods for this type. However, some studies have found that the AIRE gene is not the cause of T1DM in patients with APSI, only when merging other susceptibility genes such as 5’variable number of tandem repeat (VNTR), patients will develop T1DM.^[36]^ One female patient in this study was diagnosed with APSI. The patient had hypoparathyroidism and T1DM as the first symptom. AD and Graves disease occurred 4 years later. Laboratory tests indicated that thyroid and parathyroid related antibodies were positive, insulin, cortisol, and parathyroid hormone were decreased, and thyroid hormone levels were increased, which was consistent with the above characteristics. However, due to economic reasons, this patient did not have AIRE gene test.

APSII is the most common type of adult type, more common in women, often in the onset of adulthood, 40 to 50 years old to reach the peak incidence.^[37]^ The current pathogenesis of this type is still unclear, it is only known to be associated with polygenic genetic susceptibility. Among them, human leukocyte antigen (HLA)-DR3/DQ2 and DR4/DQ8 are most closely related to the broken arm of chromosome 6,^[38]^ and other susceptibility genes include cytotoxic T-lymphocyte-associated antigen 4 (CTLA- 4) genes, protein tyrosine phosphatase non-receptor type 22 (PTPN22) gene, forkhead box gene P3 (FOXP3), VNTR gene, major histocompatibility complex class I chain-related gene A 5.1 (MICA5.1), etc. Because of its genetic background with T1DM, T1DM has the highest incidence in this type, and about 60% of patients with APSII can be combined with T1DM.^[2]^ The most common phenotype of APSII is AITD, the incidence rate is 70% to 75%, followed by type 1 diabetes, and the incidence of AD is third, about 40% to 50%. The most common combination in the clinic is AITD combined with T1DM, accounting for about 60%, and AITD combined with AD is rare, accounting for only 14.6%.^[39]^ Because the pathogenesis of APSII is unknown, the main clinical diagnosis is based on related antibodies, endocrine gland hormone levels and clinical symptoms. Due to the high incidence of this type, it is suggested that after the first autoimmune endocrine gland disease appears, screening of thyroid, islet and adrenal related antibodies and hormone levels should be routinely screened every 3 to 5 years.^[40]^ The subjects enrolled in this study did not find APSII patients, which may be related to the regional distribution of susceptibility genes and the late onset of the disease.

APSIII and APSII are mostly the same in disease coverage and pathogenesis. Therefore, this type is often classified as APSII in clinical practice. There is little research on this single type. A Polish study of 461 patients with T1DM found that the incidence of APSIII was about 14.5%, of which APSIIIA accounted for 11.1% and APSIIIC accounted for 3.5%. The incidence was positively correlated with patient age and female gender.^[41]^ The incidence rates of APSIIIA and APSIIIC patients in this study were 23.53% and 5.88%, respectively, which were significantly higher than the above studies. It is considered to be related to high iodine diet and high incidence of hyperthyroidism in China. This study did not find the relationship between the incidence of APSIII and the age and gender of patients.

## 5 Conclusion

1. In T1DM patients, their autoimmune response is not limited to islets, and the risk of other autoimmune diseases is significantly higher than that of normal people. GADAb-positive patients or patients with more than 1 islet antibody-positive patient are at higher risk of developing other autoimmune diseases.
2. T1DM patients with APSIIIA are the most common, and the incidence of thyroid autoimmune disease is the highest, and as the patient ages, the course of disease increases, and the risk of morbidity increases.
3. Patients with T1DM should be routinely screened for thyroid hormone levels and related antibodies at the time of diagnosis and reviewed every 2 years.
4. In patients with T1DM who have other symptoms of endocrine gland dysfunction, the corresponding hormone levels should be tested immediately to achieve early detection, early treatment, and improved patient outcome.

## Author contributions

**Conceptualization:** Shuchun Chen, Yang Liu.

**Data curation:** Yang Liu, Liqin Wang.

**Funding acquisition:** Shuchun Chen.

**Investigation:** Yang Liu, Dongmei Zhang.

**Methodology:** Yang Liu, Shuchun Chen.

**Project administration:** Xing Wang, Xing Xie, Haijiao Zhu.

**Resources:** Luping Ren.

**Software:** Yang Liu, Liqin Wang.

**Supervision:** Zelin Li.

**Validation:** Xing Wang, Xing Xie, Haijiao Zhu.

**Writing – original draft:** Yang Liu.
